# Temperamental predictors of subjective well‐being from early adolescence to mid‐life: The role of temporal and energetic regulation

**DOI:** 10.1002/ijop.12414

**Published:** 2017-02-14

**Authors:** Agnieszka Bojanowska, Anna M. Zalewska

**Affiliations:** ^1^ Faculty of Social Sciences and Design in Poznan, Department of Psychology SWPS University of Social Sciences and Humanities Poznan Poland

**Keywords:** Temperament, Subjective well‐being, Temporal and energetic regulation, Satisfaction with life, Positive and Negative Affect

## Abstract

We investigated links between temperament traits described in Strelau's Regulative Theory of Temperament (Emotional Reactivity, Briskness, Activity, Endurance, Perseveration and Sensory Sensitivity) and subjective well‐being (SWB)—Positive Affect, Negative Affect and Life Satisfaction as conceptualised by Diener. Participants representing early (n = 166) and late adolescence (n = 199), early (n = 195) and mid‐adulthood (n = 156) filled out Formal Characteristics of Behaviour—Temperament Inventory, Positive and Negative Affect Schedule and Satisfaction with Life Scale. Results showed that higher Briskness, Endurance, Activity, lower Perseveration and Emotional Reactivity corresponded with higher SWB. They predicted 16% of affective components' and 7% of satisfaction variance. Each well‐being component had a unique set of predictors; however, predictors of affective components varied across age groups. Higher Positive Affect was predicted by traits responsible for energetic regulation (higher Endurance and Activity and lower Emotional Reactivity) and by higher Perseveration, but their role (excluding Emotional Reactivity) was age‐dependent. Higher Negative Affect was predicted by higher Emotional Reactivity and dimensions expressing temporal characteristics, lower Briskness and higher Perseveration (Perseveration was not significant among younger adolescents). Higher Satisfaction was steadily predicted by lower Emotional Reactivity and higher Activity. To conclude, the functions of temperament traits are mostly in line with theoretical expectations, but more complex than indicated by previous research.

Regulative Theory of Temperament (RTT, Strelau, 2008; Strelau & Zawadzki, 1995) suggests that temperament traits regulate numerous areas of everyday functioning and may be significantly associated with subjective well‐being. The significance of temperament traits for well‐being has been studied to some extent, but there is no clear overview or straightforward empirical proof for the relationship between temperament traits and happiness. In the present article we aim to show how six traits of temperament are connected to the levels of three components of subjective well‐being—Positive Affect, Negative Affect and Satisfaction with Life (Diener, 2000).

Temperament is usually defined as a set of stable characteristics of behaviour. It was originally differentiated from personality by the proportion of genetic components responsible for its variability (DeYoung & Gray, 2009). There are, however, different approaches to studying and defining temperament, mainly stemming from the Pavlovian biological approach. These conceptions stress not only the biological determinants of individual differences in behaviour but also the stylistic and energetic aspects of behaviour regulation as opposed to content‐related aspects of behaviour found in personality dimensions (Kandler, Riemann, & Angleitner, 2013). These stylistic and energetic aspects of behaviour may be significantly associated with subjective well‐being, but their specific roles for different components of subjective well‐being have not yet been empirically verified.

In this paper, we conceptualise temperament according to Strelau's RTT (Strelau, 2008; Strelau & Zawadzki, 1995). RTT refers to formal aspects of behaviour and not content‐related, concentrating on the “how” of behaviour and not the “what” and stresses energetic and temporal regulation as the core of temperament's function. We conceptualise subjective well‐being as consisting of the two affective components (Positive and Negative Affect) and the cognitive component based on evaluative beliefs (attitudes) about one's life (Life Satisfaction; Diener, 2000).

### Individual determinants of subjective well‐being

Subjective well‐being depends, to a great extent, on individual traits. Individuals have global tendencies to experience life in a positive or negative way (DeNeve & Cooper, 1998) and each person has their own happiness baseline (Brickman & Campbell, 1971). Individual tendencies and preferences determined by personal traits interact with the outside world, because they influence the interpretations of external circumstances (Zalewska, 2004). Consequently, personality and temperament are said to be one of the strongest source predictors of subjective well‐being (DeNeve & Cooper, 1998). Most of the research to date, however, focused on the role of personality and there is only little data on the role played by temperament (conceptualised as energetic regulation and stylistic characteristics of behaviour). For example, Costa and McCrae (1980) suggested that high Extraversion and low Neuroticism constitute the “happy personality”, because extraverts are more cheerful and high‐spirited in comparison to introverts, and emotional instability facilitates the experience of negative emotions. These personality traits, however, explain only part of the mechanisms responsible for stable differences in individual well‐being. Temperament as conceptualised by the RTT has a lot of potential for explaining these mechanisms at a more formal level, with focus on temporal and energetic regulation.

### RTT and subjective well‐being

Individual characteristics responsible for energetic level described in the RTT regulate all aspects of functioning, from the choice of preferred environments, including social interactions, to interpretations of stimuli (Strelau, 2008).These differences in functioning stemming from differences in temperament may in turn impact well‐being. RTT (Strelau & Zawadzki, 1995, see also a summary of the conception in Kandler et al., 2013) states that temperamental dimensions refer to formal aspects of behaviour, in that all behaviour can be described with regard to its energetic and temporal characteristics. According to RTT, there are six non‐orthogonal temperament traits, among them the first two refer to temporal characteristics of behaviour and the other to its energetic aspects:

1. Briskness (BR): tendency to react quickly, to keep a high tempo of performing activities and to shift easily in response to changes in the surroundings from one behaviour (reaction) to another;
2. Perseveration (PE)^1^: tendency to continue and to repeat behaviour after cessation of stimuli (situations) evoking this behaviour;
3. Sensory Sensitivity (SS): ability to react to sensory stimuli of low stimulative value;
4. Emotional reactivity (ER): tendency to react intensively to emotion generating stimuli, expressed in high emotional sensitivity and in low emotional Endurance;
5. Endurance (EN): ability to react adequately in situations demanding long‐lasting or high stimulative activity and under intensive external stimulation;
6. Activity (AC): tendency to undertake behaviour of high stimulative value or to supply, by means of behaviour, strong stimulation from the surroundings (Strelau, 2008; Strelau & Zawadzki, 1995).

The functions of these six traits, as well as their correlations with the Big Five, suggest that they may be connected to the experience of well‐being. Inferences may be drawn from the directions of these correlations (Strelau & Zawadzki, 1995).

### Beneficial functions of Briskness, Endurance and Activity

There is no direct empirical data on the links of these temperament traits and subjective well‐being. In order to show possible effects of these traits on subjective well‐being we must rely on data from research on psychopathology and other well‐being domains. The analyses of these studies suggest that high Briskness and Endurance may have positive effects on well‐being. The beneficial role of Briskness was demonstrated in studies on stress (Fruehstorfer, Veronie, Cremeans‐Smith, & Newberry, 2012; Zawadzki & Popiel, 2012) and the beneficial role of Endurance in studies on burnout (Cieslak, Korczynska, Strelau, & Kaczmarek, 2008).

Theoretical descriptions of Briskness, Endurance and Activity also suggest that they are beneficial. Higher Briskness means a faster pace, easier shifting between activities connected with higher plasticity and this leads to a more diverse experience. This probably facilitates higher satisfaction and Positive Affect. Higher Endurance implies that a person is able to function effectively in a wider array of circumstances, is able to resist distractions, which may lead to greater satisfaction and give more opportunities to experience positive emotions. Higher Activity may be connected to advantageous mood profile, as people with higher Activity engage in more enterprises.

### Unbeneficial functions of Emotional Reactivity and Perseveration

Perseveration seems unbeneficial for well‐being, this was demonstrated in studies on burnout (Rzeszutek & Schier, 2014) and coping with illness. High Perseveration was linked to lingering Negative Affect and poorer coping strategies (Heszen, 2012).

Emotional Reactivity got perhaps the widest attention in recent literature on quality of life. Its high levels were linked to increased stress and anxiety or decreased job satisfaction (Zalewska, 2011). One study by Bojanowska and Zalewska (2011) showed that lower Emotional Reactivity predicted higher Life Satisfaction and Positive Affect and lower Negative Affect among teenagers. There is no data on Sensory Sensitivity and its role for well‐being. The functions of this trait do not suggest specific links to well‐being.

### Temperament, age and subjective well‐being

Subjective well‐being has a specific developmental dynamic (Argyle, 1987) and so does temperament. Age groups systematically differ in temperament trait levels (Zawadzki & Strelau, 1997). Therefore, the links between these two dimensions may differ between age groups. In our study, we compare four groups, representing four periods of development: early and late adolescence, early adulthood and mid‐life. They represent distinct stages of cognitive, social and emotional development (e.g. Levinson, 1986) and the differences between these stages may moderate the functions of temperament dimensions for the dimensions of well‐being.

### General hypothesis

Since there is a dearth of research into RTTs temperament traits and subjective well‐being, we formulate the following hypothesis basing on studies on other well‐being areas and theoretical descriptions of the traits. We expect, that higher Briskness, Endurance and Activity, lower Perseveration and lower Emotional Reactivity are connected to higher well‐being indices. There is also no empirical evidence to predict specific functions of these traits for specific components of well‐being. Therefore this article also aims to answer how temperament traits predict components of subjective well‐being (Positive Affect, Negative Affect and Satisfaction). We also aim to analyse, how the possible links between temperament and well‐being hold in various age groups.

## METHODS

### Participants

Participants (*N* = 716) represented four age groups: early adolescence (13–14 years old: *n* = 166), late adolescence (17–18 years old: *n* = 199), early adulthood (28–33 years old: *n* = 195) and mid‐life (40–45 years old: *n* = 156). These four groups cover subsequent stages of development in the first “half” of life (Levinson, 1986). There was a close to even distribution of women and men (women/men: adolescents 54/46%; late adolescents 40/60%; young adults 53/47%; mid‐life 56/44%). We followed all ethical standards and the study was approved by the local ethical committee.

### Procedure

In the group of teenagers, the study was conducted in class in the presence of the teacher. Students were assured that refusing to participate would have no negative consequences, participation was voluntary and anonymous, and personalised data would not be analysed. In the adult sample, the questionnaires were distributed in the workplace (after getting permission from a manager or Human Resources Department) and a deadline for return was set. The participants were informed that the study was anonymous and voluntary. The questionnaires were placed in an envelope to be sealed before returning them and filled out within a week.

### Measures

#### *Formal characteristics of behaviour—temperament inventory*

Six temperamental traits were measured using Formal Characteristics of Behaviour—Temperament Inventory (Zawadzki & Strelau, 1997). These traits were Briskness, Perseveration, Sensory Sensitivity, Emotional Reactivity, Endurance and Activity (Strelau, 2008; Strelau & Zawadzki, 1995). There were 20 items pertaining to each trait, with yes/no answers. The level of each temperament trait was computed by summing answers to items of each trait subscale so that a higher index indicated a higher level of a particular trait: 1 point for an answer indicating a higher level and 0 points for an answer indicating a lower level of that particular trait. The sums could consequently range from 0 to 20 for each temperament trait.

#### *Well‐being indices*

Positive and Negative Affect were measured using Positive and Negative Affect Schedule (PANAS, Watson, Clark, & Tellegen, 1988) translated into Polish with a back‐translation. The measure includes a list of 10 adjectives referring to Positive (e.g. interested, excited) and 10 to Negative Affective states (e.g. guilty, ashamed) and respondents were asked to indicate how intensely they had felt this way during 2 weeks before the study. The scale ranged from 1 (only slightly or not at all) to 5 (extremely).

Satisfaction with Life was measured using Satisfaction with Life Scale (SWLS, Diener, Emmons, Larsen, & Griffin, 1985). Participants indicated to what extent they agreed with the statements about their lives (e.g. in most ways my life is close to my ideal) on a scale from 1 (I definitely disagree) to 7 (I definitely agree). Higher scores meant higher Life Satisfaction.

### Data analyses

To test whether higher Briskness, Endurance and Activity, lower Perseveration and lower Emotional Reactivity are connected to higher subjective well‐being dimensions (higher Positive Affect and Satisfaction and lower Negative Affect), we used the Pearson product–moment correlation coefficients. To answer how temperament traits predict subjective well‐being components, we conducted Structural Equation Modeling (SEM) analysis using AMOS graphics. We created an initial model with three well‐being components as endogenous variables and six temperament traits as exogenous variables, intercorrelations between temperament traits, as well as intercorrelations between residual terms of endogenous variables (insignificant paths were later removed). This analysis allowed us to determine specific predictive value of each temperament trait while controlling for the influence of the other temperament dimensions. Analogically, we conducted multigroup SEM to test the same model (with the same variable configuration) across the four age groups.

## RESULTS

### Descriptive statistics and intercorrelations

Table 1 displays descriptive statistics of the main variables. All scales demonstrated sufficient reliabilities. Apart from Sensory Sensitivity, all variables are weakly to moderately correlated and the directions of “zero‐order” correlation coefficients have been fully consistent with the hypothesis: higher Briskness, Endurance and Activity, lower Perseveration and lower Emotional Reactivity are connected to higher well‐being.

**Table 1:** Means, standard deviations, internal consistencies and intercorrelations between well‐being indices and temperament traits

					Intercorrelations								
		M	SD	α	PA	NA	S	BR	PE	SS	ER	EN	AC
Positive Affect	PA	3.31	.67	.81	−	−.13***	.36***	.25***	−.09*	.02	−.31***	.30***	.29***
Negative Affect	NA	2.17	.76	.86		—	−.33***	−.27***	.31***	−.02	.38***	−.26***	−.13***
Satisfaction	S	4.40	1.14	.82			—	.18***	−.12***	.01	−.24***	.13**	.18***
Briskness	BR	14.50	3.96	.79				—	−.28***	.20***	−.47***	.45***	.32***
Perseveration	PE	12.76	4.24	.76					—	.21***	.63***	−.39***	−.18***
Sensory Sensitivity	SS	14.22	3.76	.76						—	.04	−.07	.07
Emotional reactivity	ER	10.63	4.76	.81							—	−.58***	−.40***
Endurance	EN	10.51	4.83	.85								—	.21***
Activity	AC	9.68	4.72	.82									—

* *p* < .05.

** *p* < .01.

*** *p* < .001.

### Temperament traits as predictors of subjective well‐being

**Figure 1:**
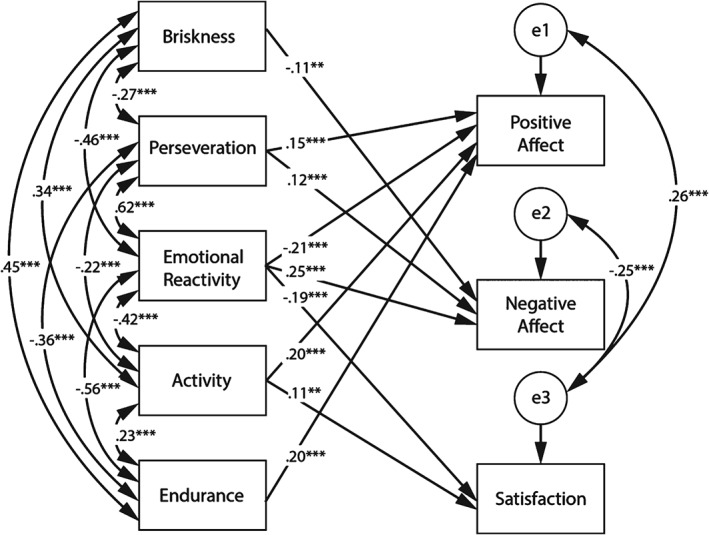
The model of relationships between subjective well‐being and temperament traits with standardised path coefficients (* p < .05. ** p < .01. *** p < .001).

The model of relationships between three well‐being components (Positive and Negative Affect and Satisfaction) and temperament traits (Emotional Reactivity, Activity, Briskness, Endurance and Perseveration) is shown in Figure 1. After drawing the initial model, we computed estimates, removed insignificant paths and analysed fit indices. This resulted in full removal of Sensory Sensitivity from the model, because it was not significantly linked to any of the well‐being indices. Figure 1 presents regression weights for estimates in the model paths.

The model fit was evaluated based on three goodness of fit indices, provided by the software and discussed by Wu and Lei (2007): (*χ* ^2^ [*df* = 7, *N* = 716] = 5,90; *p* > .05,/Tucker Lewis index (TLI = .99), the comparative fit index (CFI = .99) and the root mean square error of approximation (RMSEA = .03) indicated good fit and are correctly placed in reference to cutoff points suggested by Wu and Lei (TLI, CFI > .95, RMSEA < .06).

Each component of subjective well‐being was predicted by a different set of significant predictors. Higher Perseveration, Activity and Endurance and lower Emotional Reactivity predicted more intense Positive Affect and these predictors explained 16% of Positive Affect variance. Higher Negative Affect was predicted by lower Briskness and higher Perseveration and higher Emotional Reactivity (16% of variance explained). Higher Satisfaction was predicted by lower Emotional Reactivity and higher Activity (7% of variance explained).

### Comparison of age groups

We compared the model of relationships between temperament dimensions and subjective well‐being indices across age groups (multigroup SEM). First, we removed paths insignificant in all groups, which resulted in a model identical to the one tested for the whole group (Figure 1). Then, we compared each effect between these groups. Only some effects differed between groups, most effects held for all groups. Table 2 presents these comparisons (only data for significant differences are reported). The role of Perseveration for both affects was not significant only among younger adolescents, so was the role of Activity for Positive Affect. Positive Affect, however, was linked to Endurance only among adolescent groups.

**Table 2:** Effects of temperament dimensions for Positive Affect, Negative Affect and Satisfaction with Life in early and late adolescence, early adulthood and mid‐life. Results of multigroup structural equation modelling. Comparison of critical ratios for group differences (z‐statistic)

	Early adolescence^a^	Late adolescence^b^	Early adulthood^c^	Mid‐life^d^	Significant differences in effects (*z*‐statistics for pairs of groups)
Perseveration →Positive Affect	.05	.28***	.16ˆ	.17ˆ	1.97** ^ab^
Perseveration →Negative Affect	−.08	.17*	.15ˆ	.14ˆ	2.14** ^ab^
Activity→Positive Affect	.03	.25***	.17*	.34***	1.20* ^ab^, 2.43** ^ad^
Endurance→Positive Affect	.29***	.20*	.06	.15	−2.15** ^ac^

*Note*: Alphabets in superscript indicate groups that differ significantly.

ˆ *p* < .1.

* *p* < .05.

** *p* < .01.

*** *p* < .001.

We also analysed age differences in the levels of variables. These comparisons are shown in Table 3. These differences in temperament traits are consistent with patterns detected in validity studies (Zawadzki & Strelau, 1997).

**Table 3:** Means, standard deviations and analysis of variance comparisons across age groups

	Early adolescence^a^	Late adolescence^b^	Early adulthood^c^	Late adulthood^d^	Significant differences for pairs of groups	
	M (SD)	M (SD)	M (SD)	M (SD)	F (df = 3712)	
Perseveration	12.94(3.61)	14.18(4.00)	11.72(4.51)	12.06(4.20)	13.61***	c < a; b > acd
Endurance	11.56(3.96)	9.43(4.71)	10.75(4.84)	10.50 (5.43)	6.23***	a > bd; b < cd
Activity	9.68(4.44)	10.96 (4.43)	9.85 (4.64)	7.73(4.87)	14.66***	d < abc, b > ac
Emotional Reactivity	10.90(4.08)	11.40(4.41)	9.67(5.05)	10.58(5.37)	4.59**	c < ab
Briskness	13.50(3.68)	14.32(3.60)	15.05(3.61)	15.07(4.62)	6.26***	a < bcd
Positive Affect	3.38(.67)	3.21(.70)	3.43(.68)	3.27(.60)	4.37**	a > b, c > bd
Negative Affect	2.11(.74)	2.27(.70)	2.21(.79)	2.04 (.79)	3.33*	a < b, bc > d
Satisfaction	4.58(1.21)	4.28(1.05)	4.49(1.13)	4.39(1.12)	2.45	

*Note*: Alphabets in superscript indicate groups that differ significantly.

* *p* < .05.

** *p* < .01.

*** *p* < 0.001.

## DISCUSSION

In the present study we analysed the relationships between subjective well‐being as conceptualised by (Diener, 2000; Positive Affect, Negative Affect and Satisfaction with Life) and six temperamental traits proposed in RTT (Strelau & Zawadzki, 1995; Briskness, Perseveration, Sensory Sensitivity, Emotional Reactivity, Activity and Endurance).

### Can temperament predict subjective well‐being?

As expected, subjective well‐being components (higher Life Satisfaction and Positive Affect and lower Negative Affect) were correlated with five temperament traits: higher Briskness, Endurance and Activity and lower Perseveration and Emotional Reactivity. These results of “zero‐order” correlations are fully consistent with what we know about relationships between temperament, personality and subjective well‐being. Our results are also concordant with earlier findings about correlations between particular temperament dimensions and specific negative aspects of well‐being (Cieslak et al., 2008; Fruehstorfer et al., 2012; Rzeszutek & Schier, 2014; Zawadzki & Popiel, 2012). However, we found more effects than could be directly inferred from previous studies on personality and temperament or temperament traits' definitions. All components of subjective well‐being were related to five temperament traits (with the exception of Sensory Sensitivity), but their roles differed when the whole set of traits was included in one analysis (to control for intercorrelations between traits).

Our study indicates that temperament is more strongly linked to affective component of subjective well‐being than to satisfaction with life. This is congruent with the claim that personality and other individual characteristics mostly influence the affective component (Schimmack, Schupp, & Wagner, 2008; Zalewska, 2003). However, we also found that not only does the percentage of explained variances differ, but that each component of subjective well‐being has its own unique set of predictors. This indicates that temperamental functions for well‐being components are complex. Furthermore, we found that the links between temperament and well‐being vary across age groups.

The directions of the influence cannot be directly inferred from this study. Yet, RTT stresses the regulative role of temperament traits, so we assume, that they may facilitate or hamper well‐being. This supposition needs to be verified in further studies.

### Functions of temperament traits

Firstly, high *Emotional Reactivity* is linked to lower subjective well‐being. People with lower Emotional Reactivity are more emotionally stable and they tend not to interpret stimuli in emotional terms (Strelau, 2008). Their emotional interpretations of ambiguous stimuli are also more positive and they tend to react with less negative emotions as compared to highly emotionally reactive individuals (Zalewska, 2003, 2011). High Emotional Reactivity is linked to poorer well‐being (in all three components), possibly through a decreased effectiveness in functioning under stress, leading to less effective and less successful outcomes of individual activities (Strelau, 2008). Functions of Emotional Reactivity include performance in stress, so through a decreased performance, more reactive people may experience not only more negative emotions, but also less positive emotions, evaluate their lives as less satisfying in comparison to less reactive people, who perform better in a wider array of circumstances (Strelau, 2008).

Secondly, higher *Perseveration* was linked to stronger positive and negative affect, but these effects were not found among younger adolescents. This suggest, that from late adolescence the core function of Perseveration (tendency to continue and to repeat behaviour after the stimulus ceased, Strelau & Zawadzki, 1995) refers not only to behaviour but also to accompanying emotions. The role of Perseveration is therefore not straightforward, it is connected to more intense negative and positive emotions, in other words: a more diverse emotional experience. This suggests that high Perseveration among adults and older adolescents can also serve a beneficial function—it may promote the experience of positive emotions, when the influence of other temperament traits has been controlled for. In these groups, Perseveration may be necessary for prolonged experience of positive emotions (Positive Affect)—without it Positive Affect is vanishing very quickly. On the other hand, it may also prolong the experience of negative emotions, serving a rather unbeneficial function.

Thirdly, higher levels of *Activity* were connected to higher Positive Affect and Satisfaction. This means that positive indices of subjective well‐being, higher Life Satisfaction and Positive Affect are possibly facilitated by Activity, but this trait may not limit the experience of negative emotions. The Activity‐Positive Affect link, however, did not hold in the youngest group. Possibly, Activity in this youngest group is still regulated by other people (parents, teachers) and therefore its positive role emerges only later, when teenagers start regulating their activities on their own.

Fourthly, *Briskness* is the trait responsible for the temporal regulation of behaviour and it had specific, limited effect: lower Briskness predicted stronger Negative Affect. This suggests that the inability to shift quickly between tasks may result in frustrations with ineffective resource management.

Finally, *Endurance* was only linked to the Positive Affect, and only among adolescents. According to the definition, highly enduring individuals process stimulation more efficiently, so they can experience Positive Affect even when faced with more stimulation. We confirmed this result in two groups of adolescents and it can be explained by different Endurance scores across age groups: younger adolescents had the highest Endurance scores and older adolescents had the lowest Endurance scores. Possibly, the role of this trait is only significant with high or low scores. Endurance is probably significant even if the Activity is not voluntary—such as may happen among younger adolescents. In this youngest group, Activity and Positive Affect were not linked, but Endurance and Positive Affect were. Nevertheless, teenagers' low Activity combined with high Endurance may produce relatively high levels of Positive Affect. On the other hand, older adolescents' lower Endurance seemed particularly important and unbeneficial, because it was accompanied by high Activity. This may lead to excessive stimulation and be reflected in lowered Positive Affect. Moreover, low scores in Endurance may prevent older adolescents from engaging in strenuous tasks, therefore limiting their chances for activities evoking positive emotions. To sum up, the effect of Endurance, seems to be limited only to adolescents, either due to specific developmental characteristics, or due to their Endurance scores.

### Temperamental predictors of Positive Affect among adolescents and adults

Well‐being components varied in their sensitivity to age group comparisons. Temperament‐Satisfaction links were similar across age groups. For the Negative Affect the only group difference referred to the role of Perseveration—this trait was not linked to negative emotions only among younger adolescents. In reference to Positive Affect, the links differed strongly across age groups (for Endurance, Perseveration and Activity).

In general, higher Positive Affect was predicted by higher Activity, Endurance and Perseveration, and lower Emotional Reactivity. Three of these traits (excluding Perseveration) determine effectiveness of stimulation regulation. When Endurance is higher, and Emotional Reactivity is lower, a person is less susceptible to tiredness and distractions, more resistant to emotional stimuli, able to process a lot of stimulation and achieve high results without physiological cost. When these trait levels are accompanied by higher Activity, a person can regulate behaviour according to higher processing capacities—engage in numerous and demanding activities and withstand a lot of stimulation. Then the cost of high Activity is low and the benefits are high. Low Emotional Reactivity, high Endurance and Activity allow to achieve and maintain the optimal level of arousal (activation), which results in high Positive Affect. In other words higher Activity, Endurance and lower Emotional Reactivity may promote the experience of positive emotions through more effective regulation, and additionally higher Perseveration helps prolong the experience of these emotions.

These effects have not been detected in earlier studies, mostly because researchers focused on the links between temperament and negative aspects of well‐being, such as burnout, stress management and somatic illness (Cieslak et al., 2008; Rzeszutek & Schier, 2014). Investigations of temperamental predictors of positive affectivity were rare or non‐existent and data on their mutual relationships were scarce.

This pattern was expressed in full only among older adolescents. Among younger adolescents, the role of Activity and Perseveration were not significant, only Emotional Reactivity and Endurance remained, and these are the two traits that together determine stimulation processing capacities. Among adults Positive Affect was predicted by higher Perseveration, lower Emotional Reactivity and higher Activity—the two last traits most strongly express the harmony between the amount of stimulation supplied and the ability to process it and they are most strongly linked to Neuroticism and Extraversion (Costa & McCrae, 1980; Zawadzki & Strelau, 1997).

Naturally, these effects can be cohort‐related since this is a cross‐section study. If, however, they are related to developmental changes, then the study suggests that the period of development moderates the links between temperament and well‐being. Among younger adolescents, high Positive Affect was predicted only by traits responsible for high stimulation processing capacity (high Endurance and low Emotional Reactivity), possibly because their activity is more externally controlled. Among older adolescents, it was predicted by traits responsible for effective stimulation regulation—connected with stimulation processing capacities (Emotional Reactivity and Endurance), stimulation supply (Activity) and additionally by high Perseveration, which may help prolong the experience of positive emotions. Among adults, the role of traits expressing stimulation processing capacity seems to become relatively less important in predicting the Positive Affect, as Endurance is no longer its significant predictor. All this suggest that temperamental regulatory functions may be expressed differently, depending on the period of development—at least for the Positive Affect.

### Temperamental predictors of Negative Affect

In general, higher Negative Affect was predicted by lower Briskness, higher Perseveration and higher Emotional Reactivity; however, among younger adolescents Perseveration was not a significant predictor. Higher Emotional Reactivity determines a stronger tendency to react intensely to emotional stimuli, expressed in higher emotional sensitivity and lower emotional resistance, this means (by definition) higher susceptibility to stress and negative emotions. Two remaining traits (Perseveration and Briskness) are responsible for the temporal regulation of behaviour. Lower Briskness may increase Negative Affect through the inability to react quickly, keep a sufficient tempo of performance, and inability to shift in response to changes in the surroundings from one behaviour (reaction) to another (Zawadzki & Strelau, 1997). Lower Briskness may lead to ineffective behaviours and slower adaptation to changing circumstances, the cost of which are manifested in the experience of negative emotions. Additionally, among adults and older adolescents higher Perseveration may facilitate higher Negative Affect through the tendency to continue and to repeat behaviour after the stimulus evoking this behaviour ceased to act. This continued behaviour may be associated with negative emotions if the Activity is no longer effective or needed because the circumstances have changed. A person with higher Perseveration may need time to cease behaviour or suppress emotions evoked earlier. In other words, higher Briskness may help launch new behaviours when needed, it seems to have an adaptive role in all age groups, while lower Perseveration may have the potential to shield from Negative Affect, but only in some age groups.

### Temperamental predictors of Life Satisfaction

Only higher Life Satisfaction was steadily predicted by lower Emotional Reactivity and higher Activity. These traits are most strongly linked to Neuroticism and Extraversion, that is the two components of the “happy personality” (Costa & McCrae, 1980; Zawadzki & Strelau, 1997). The insignificant predictive value of Endurance indicates that higher Activity and lower Emotional Reactivity are sufficient for achieving higher Life Satisfaction. This means that individuals with such trait combination may be more satisfied even if they achieve satisfaction at a physiological cost (e.g. tiredness) or at the cost of making mistakes caused by distractions.

### Limitations

In this study, the sample was entirely Polish so the detected effects might be limited to the Polish society. Possibly, the understanding of happiness and subjective well‐being may be culture specific (Bojanowska & Zalewska, 2016a; Delle Fave, Brdar, Freire, Vella‐Brodrick, & Wissing, 2011), but we expect that at least some of these effects may be universal. Secondly, the study covered only the first “half” of life, so some of the effects might pertain only to younger people. With the developmental dynamic of temperament, it is possible that as trait levels change with age, so do their functions for well‐being. Thirdly, this study relies on cross‐sectional data, so we are unable to determine the direction of influence—we cannot say whether temperament influences well‐being or whether these two aspects of human properties share common determinants (and variance). We make claims about the mechanisms underlying these links, but they are mainly hypothesised functions stemming from the theoretical assumptions of the RTT.

## CONCLUSIONS

In the present study, we showed that five of six temperament traits included in RTT (excluding Sensory Sensitivity) are correlated with all components of well‐being. Their relationships, however, proved to be more complex when the whole set of traits was controlled for. Temperament traits more strongly predicted affective components than satisfaction. Each well‐being dimension had a unique set of predictors, but these sets for the affective components were not universal—they depended on age group. These differences in SWB predictions were most pronounced for the Positive Affect, suggesting that positive emotions may be facilitated or hampered by unique characteristics and conditions. They show that energetic and temporal regulation is significant for the experience of well‐being and that relationships between temperament traits and SWB components are more complex than could be expected from previous research on temperament–SWB and personality–SWB relations. Previous temperament–SWB investigations focused mainly on negative aspects of well‐being, neglecting its possible roles for the positive aspects of human experience. Personality–SWB research, on the other hand, focused mostly on the role of Neuroticism and Extraversion (Costa & McCrae, 1980; DeNeve & Cooper, 1998), which suggested how Emotional Reactivity and Activity may impact SWB, but did not offer such explanations for Perseveration, Endurance or Briskness. In our study, we showed that a clear correspondence to effects of personality were only found for Satisfaction—it was predicted by Emotional Reactivity (overlapping functions with Neuroticism) and Activity (overlapping functions with Extraversion). The other components had additional predictors, whose functions could not be simply detected in personality research, because they are not discussed in personality theories.

Although these data do not allow for conclusions about causality, the assumptions of the RTT, combined with our results, suggest that each trait has a unique function for subjective well‐being. However, this may need to be studied further in terms of causality and also in terms of specific configurations of traits levels. As stated in the RTT, some traits can be clustered together and these clusters may interact with one another to produce harmonious or disharmonious temperament types. This suggests that a typological (as opposed to trait‐oriented) approach may be required to better explain how interactions between these traits relate to subjective well‐being (Bojanowska & Zalewska, 2016b). Finally, there is a need to analyse the age differences with greater detail. We showed that some of these mechanisms may not be entirely universal—temperament may predispose people for specific well‐being experience—but there may be other mechanisms impacting these relationships, such as those related to learning and individual development.

## Footnotes

1 In original publication, Strelau and Zawadzki (1995) used the term “Perseverance,” but it caused some misunderstandings, so in more recent publications the term “Perseveration” is used (Strelau, 2008).
